# Supplementary material for: Co-Designing an Inclusive Stakeholder Engagement Strategy for Rehabilitation Technology Training Using the I-STEM Model
Source: Int J Environ Res Public Health. 2025 Dec 20;23(1):13. doi: 10.3390/ijerph23010013 (PMC12841479; doi:10.3390/ijerph23010013)
Supplement: Supplementary file 1 [file ijerph-23-00013-s001.zip › ijerph-3987452-supplementary/ijerph-3987452-supplementary material/S1 Table.pdf]

S1 Table. Use of GRIPP2-SF to reflect on PPI

GRIPP2-SF section	Description
Aim	To determine stakeholder views on (a) factors related to the inclusivity and accessibility of training on rehabilitation technologies to individuals with sensory, cognitive and physical impairments, and (b) patient needs associated with training in rehabilitation technologies.
Methods	Contributions were gathered via asynchronous (email, text, survey, video) and synchronous (meetings, calls) formats. We recruited 15 individuals (6 rehabilitation professionals, 5 patients, 2 health educators and 2 innovators), via professional networks and local support groups.
Results	Their input established the need for education and training in rehabilitation technologies and increased our understanding of the key challenges for all stakeholders. This PPI led to a clear description of (a) the factors supporting inclusive and accessible patient-facing training, (b) the specific needs of rehabilitation patients related to receiving and engaging with training in rehabilitation technologies.
Discussion/ Conclusions	This PPI led to the co-creation of a stakeholder engagement plan to gather more in-depth insights on education and training needs across the stakeholder groups. The plan included education and training needs assessment and priority-setting, through an open survey with rehabilitation professionals and stakeholder consultation events with all stakeholder groups. The PPI helped to ensure that the planned research and engagement activities were user-friendly and aligned with stakeholder priorities.
Reflections/ Critical Perspective	The PPI was valuable and the flexible engagement formats were appreciated. Challenges included scheduling meetings that accommodated all contributors and ensuring diverse representation.
