# Supplementary material for: Co-Designing an Inclusive Stakeholder Engagement Strategy for Rehabilitation Technology Training Using the I-STEM Model
Source: Int J Environ Res Public Health. 2025 Dec 20;23(1):13. doi: 10.3390/ijerph23010013 (PMC12841479; doi:10.3390/ijerph23010013)
Supplement: Supplementary file 1 [file ijerph-23-00013-s001.zip › ijerph-3987452-supplementary/ijerph-3987452-supplementary material/S1 Text.pdf]

PPI Involvement Evaluation Survey

Thank you for participating in this PPI consultation. Your feedback will help us improve future activity. This survey focuses on three areas: ease of contribution, accessibility, and safety/support.

Section 1: Ease of Contribution

1. I felt my contributions were listened to and valued.

☐ Strongly Agree ☐ Agree ☐ Neutral ☐ Disagree ☐ Strongly Disagree

2. There were different ways to contribute that worked for me.

☐ Yes ☐ No ☐ Partly

3. What helped you feel comfortable contributing?

____

____

4. What made it harder to contribute?

____

____

Section 2: Accessibility

5. It was easy for me to share my views in a way that suited me

☐ Strongly Agree ☐ Agree ☐ Neutral ☐ Disagree ☐ Strongly Disagree

6. I had the support I needed to participate fully.

☐ Strongly Agree ☐ Agree ☐ Neutral ☐ Disagree ☐ Strongly Disagree

7. What, if anything, made participation difficult?

____

____

Section 3: Safe & Supportive Environment

8. I felt comfortable sharing my views without fear of judgment.

☐ Strongly Agree ☐ Agree ☐ Neutral ☐ Disagree ☐ Strongly Disagree

9. The event felt inclusive and respectful of different perspectives.

☐ Strongly Agree ☐ Agree ☐ Neutral ☐ Disagree ☐ Strongly Disagree

10. Describe three words about how this space felt for you.

____

____

____

11. What could have made you feel safer or more supported?

____

____

Thank you!

Your feedback is important to us. Please return this survey to the organisers. We will share a summary of feedback and how it will be used to improve future events.
