# Supplementary material for: Co-Designing an Inclusive Stakeholder Engagement Strategy for Rehabilitation Technology Training Using the I-STEM Model
Source: Int J Environ Res Public Health. 2025 Dec 20;23(1):13. doi: 10.3390/ijerph23010013 (PMC12841479; doi:10.3390/ijerph23010013)
Supplement: Supplementary file 1 [file ijerph-23-00013-s001.zip › ijerph-3987452-supplementary/ijerph-3987452-supplementary material/S2 Text.pdf]

NIHR | HealthTech Research Centre Rehabilitation

Stakeholder Engagement Strategy

Mapped to the Implementation STakeholder Engagement Model – I-STEM.

Project title
Determining Education and Training Needs for Rehabilitation Technologies.

Project Aim
This project seeks to identify and address the education and training needs of three key stakeholder groups - rehabilitation professionals, patients, and technology innovators - to enhance the effective adoption and use of rehabilitation technologies (e.g. assistive robotics, digital rehabilitation tools, and wearable sensors). The strategy applies the Implementation STakeholder Engagement Model (I-STEM) to ensure systematic, inclusive, and evidence-informed engagement throughout the research and implementation cycle. I-STEM offers a robust framework to ensure stakeholders are not just consulted - but actively engaged - in every step of the journey.

1. Engagement Objectives (I-STEM Step 1)

The 4 core I-STEM objectives are around understanding, verifying, and co-creating training needs.

- *Understand*: To explore and document the education and training needs, barriers, facilitators, and priorities of each stakeholder group relating to rehabilitation technologies. Engage in PPI activity and recruit a stakeholder champion ('PPI-partner') who will engage with all stages.
- *Verify*: Engage rehabilitation professionals, patients, and technology innovators to articulate and prioritise their training and education needs in using rehabilitation technologies.
  Validate and prioritise identified needs through stakeholder review and consensus-building activities. Test and refine assumptions about training needs, priorities, and delivery modalities (e.g., in-person, e-learning, mentoring) with stakeholders.
- *Do / Enrol*: Co-design priority training modules with stakeholder input to ensure relevance, usability, and acceptability. Engage stakeholders as co-design partners and champions in developing a training plan.
- *Access*: Work in collaboration with PPI-partner and the Rehab HealthTech team to facilitate access to stakeholders.

2. Stakeholder Identification (I-STEM Step 2)

2.1. Stakeholder Groups and Types

Core stakeholders include:

• *Rehabilitation professionals*: Health or care professionals working in a rehabilitation setting (i.e., any place or service that provides support to help individuals recover from illness, injury, or disability and improve their physical, mental, and social abilities). May include but is not limited to: Physiotherapist, Occupational Therapist, Speech and Language Therapist, Psychologist, Psychology Assistant, Prosthetist/Orthotist, Nurse, Mental Health Nurse, Advanced Clinical Practitioner, Dietician, Social Worker, Doctor of Rehabilitation Medicine, Pharmacist, Pharmacist Technician, Rehabilitation Engineer, Healthcare Scientist, Rehabilitation Assistant/Practitioner, Healthcare Assistant, Speech and Language Therapy Assistant, Wellbeing Officer, Case Manager.

• *Patients and carers*: People with different levels/types of disability (i.e., patients recovering from physical, neurological, or cognitive impairments). May include but is not limited to neurological conditions (e.g., stroke, spinal cord injury, traumatic brain injury), musculoskeletal and orthopaedic (e.g., post-surgical recovery, chronic pain or arthritis, mobility impairments, cerebral palsy or multiple sclerosis, amputees), ageing population (e.g., older adults with balance or gait issues), speech and cognitive rehabilitation (e.g., aphasia, cognitive decline). Advocacy representatives (e.g., carers, partners, family members, patient advocacy organisations).

• *Technology innovators*: Includes innovators, developers and suppliers. May include but is not limited to startups and SMEs in rehabilitation tech, engineers, software developers and designers, industry / medical device firms, regulatory / standards experts, health IT vendors.

Additional stakeholders with supporting or enabling roles could include:
Educators and trainers in academic institutions, hospital / clinic administrators, policy makers / funders, health IT / informatics teams, procurement professionals.

Supporting stakeholders include:
Health service managers, health educators, funders, and regulatory representatives.

2.2. Prioritisation and mapping criteria

I-STEM recommends considering six “engagement reasons” when selecting which stakeholders to engage (influence, expertise, orientation, impact, capacity, trust).

Stakeholder sub-groups are mapped against these dimensions:

Stakeholder / subgroup	Influence	Expertise	Orientation	Impact	Capacity	Trust / relationship
Senior rehabilitation clinicians / clinical leads	High	Moderate to High	May be neutral or skeptical	High (they influence adoption)	Moderate	Some trust via clinical networks
Frontline therapists	Moderate	High (practical experience)	Mixed (open to new tech)	High (they deliver rehab)	Variable	Likely good
Patients / users	Low to moderate institutional influence	High (lived experience)	Mixed orientation	High (they are end users)	Variable (time / mobility constraints)	Possibly cautious
Innovators / developers	Moderate influence	High technical expertise	Likely favorable orientation	Moderate (they deliver tools)	Generally good capacity	May not have trust in clinical domain
Educators / trainers	Moderate influence	High pedagogic expertise	Possibly favorable	Moderate to High (they deliver training)	Good	Reasonable trust
Administrators / procurement	High influence over adoption	Moderate domain expertise	Possibly skeptical or cost-driven	High	Moderate	Varies

From this mapping, we may prioritise:

- Frontline therapists (because their training uptake is critical)
- A sample of patients representing different impairment types
- A sample of tech innovators or SMEs (especially new entrants)
- Health educators in universities or vocational training (because they have high pedagogic knowledge)
- Clinic IT / infrastructure leads or procurement staff

Not all subgroups need to be engaged equally – a tiered approach can be used (core vs peripheral). Stakeholder capacity to engage must be considered (some will have limited time or less ability to travel).

Stakeholder "profiles" will be recorded in an I-STEM worksheet (name, role, interest, capacity, potential contribution).

3. Engagement Approaches and Strategies (I-STEM Steps 3–4)

Engagement approaches are mapped to I-STEM: Assess, Consult, Collaborate, Support, Disseminate, Advocate.

Assess* - Gathering information from stakeholder groups to establish the need and identify information that will inform the stakeholder engagement strategy.

Consult* – Consult with rehabilitation professionals, patients and technology innovators to explore views, experience, barriers and facilitator, and establish training priorities relating to rehabilitation technologies.

Collaborate* – Work with PPI-partner and stakeholder advisory group to analyse PPI data (production of PPI summary), interpret stakeholder consultation outcomes, and co-design training priorities plan.

Support – Identify resources (e.g., small pilot funding, technical or pedagogical support) to help stakeholders to trial or test training.

Disseminate* - Share findings from PPI activity and stakeholder consultation, through journal articles, conferences outputs, and knowledge-exchange activities to reach a wider stakeholder audience. Co-author publications together with PPI-partner.

Advocate - Identify champions who will promote prioritised training or influence adoption.

*priority for end of year 1.

4. Engagement Strategies

This documents the plan for operationalising each approach using implementation strategies.

4.1. For Assess:

Purpose: To understand stakeholder needs, preferences, and potential barriers before deeper engagement begins.

- **Conduct needs assessments.** Through PPI activities with rehabilitation professionals, patients and technology. Approaches include email correspondence, telephone calls, group discussions – at contributor preference). Provides a clear understanding of who should be engaged, how, and why – information collected informs the stakeholder engagement strategy.
  A scoping review of the training needs of health and care professionals in rehabilitation technologies will be undertaken, co-authored by our PPI-partner. Provides knowledge of published evidence. Work with PPI-partner to analyse PPI data (production of PPI summary) and interpretation of stakeholder consultation outcomes.
- **Conduct audit of engagement process.** Collect PPI and stakeholder diversity data, and evaluation survey data for all activities. Stakeholders feel heard and valued, and their insights help refine and improve implementation plans.
- **Provide feedback**: Summaries of the PPI activity will be shared to prompt reflection.

4.2. For Consult / Collaborate:

Purpose: To seek stakeholder input from the three groups on use of rehabilitation technologies, barriers, and priorities for education and training needs, and delivery approaches.

- **Conduct prioritisation activities.** To identify current use of rehabilitation technologies, to assess for readiness and identify barriers / facilitators, and to identify priority training areas and delivery approaches.
  Methods include (a) national open online survey with rehabilitation professionals, and (b) stakeholder consultation activities with rehabilitation professionals, patients and technology innovators.

  Consultation will establish training priorities and be undertaken through focus groups and workshops (in-person or virtual), with other methods of communication (e.g., individual interviews, telephone or email correspondence) included at stakeholder preference.
- **Involve a PPI-partner and advisory group**: Create an advisory group composed of representatives from each stakeholder category. To review and advise on survey content, stakeholder consultation materials, and training priorities plan resulting from the consultation events.
- **Organise co-design consensus meeting**: host a joint workshop (in person or virtual) where stakeholders from all three groups (professionals, patients,

innovators) align on priority topics. Use consensus methodology such as nominal group technique. Aim is to instil stronger ownership, deeper trust, and more sustainable implementation outcomes through shared responsibility.
- **Identify training champions**: identify 'early adopters' among the groups who will help to disseminate training priority plans and engage with future training provisions.
- **Conduct audit of engagement process.** Collect stakeholder diversity data, and evaluation survey data for all activities. Stakeholders feel heard and valued, and their insights help refine and improve implementation plans.
- **Provide feedback**: Summaries of the stakeholder activity will be shared to prompt reflection.

4.3. For Support:

Purpose: To engage early implementers. Identifying resources will occur as part of this engagement strategy (year 1). All other activities are recommendations for future engagement as training provisions are developed and/or become available.

- **Identify resources** (e.g., small pilot funding, technical or pedagogical support) to help stakeholders to trial or test training in rehabilitation technologies.
- **Train-the-trainer strategies**: once draft modules exist, train selected stakeholders (e.g. therapists, educators) to deliver them.
- **Provide ongoing training / education outreach visits**: support stakeholders via site visits, mentorship, drop-in sessions.
- **Provide local technical assistance or implementation support**: help stakeholders tailor training locally, troubleshoot technology.
- **Use a learning collaborative**: bring together early participants to share experiences, lessons, and improvements.
- **Obtain formal commitments**: secure signed agreements from clinics or education institutions to pilot or adopt training.

4.4. For Disseminate / Advocate:

Purpose: To prompt reflection and action among health educators, NIHR HealthTech Centre for Rehabilitation and partner organisations, rehabilitation professionals, patients and technology innovators. To ensure that stakeholders feel heard and valued, and that their insights have helped to refine and improve implementation plans.

- **Submit conference abstract(s) and journal article(s).** Reporting on each stage of the proposed activity. Co-author outputs with PPI-partner.

- **Engage PPI-partner and identify champions**. To support dissemination to relevant stakeholder populations. Engage respected clinicians and/or patient advocates to champion the effort.
- **Distribute training priorities**: share information through presentations, summaries, infographics, and newsletters to stakeholder communities.
- **Increase demand**: publicise benefits and potential gains of education in rehabilitation technology among stakeholder groups to generate interest.

5. Engagement Plan (I-STEM Step 5)

I-STEM recommends detailing who, what, where, when, how much, and any local adaptation. A **6-Month Phased Engagement Plan (I-STEM Step 5: Engage)** will guide engagement activities (see Table).

Phase / Month	Engagement Stage & Purpose	Who (Stakeholders)	What (Activities)	Where (Setting)	When / Duration	How Much (Resources / Effort)	Local Adaptation
Month 1 – "Assess"	Understand stakeholder needs, preferences, and barriers before deeper engagement	• PPI partner<br>• Rehabilitation professionals<br>• Patients<br>• Technology innovators<br>• Research team	• Conduct targeted needs assessments via PPI discussions, email, and calls.<br>• Develop a scoping review protocol on training needs of rehabilitation professionals (co-authored with PPI partner).<br>• Audit current engagement data (diversity, reach).<br>• Share initial PPI summaries.	Remote (online calls, email) + In-person local discussions if feasible	4 weeks (Weeks 1–4)	• research staff for coordination<br>• PPI partner time<br>• Data collection costs	Tailor contact methods (e.g. telephone vs. email) per participant preference.<br><br>Use accessible materials and language for patients.
Month 2–3 – "Consult / Collaborate" (Phase 1)	Seek stakeholder input on use, barriers, and priorities for training in rehabilitation technologies	• Rehabilitation professionals<br>• Patients<br>• Technology innovators<br>• PPI partner<br>• Advisory group	• Launch national online survey to identify current technology use and training needs.<br>• Recruit advisory group (2-3 reps per stakeholder category).<br>• Conduct group consultations (focus groups/workshops) to explore barriers/facilitators.	Virtual (Teams) and in-person at local NHS Trust, University or partner organisation meeting rooms	8 weeks (Weeks 5–12)	• Survey hosting<br>• Facilitators<br>• Vouchers for participants<br>• Transcription costs	Allow hybrid participation (virtual/in-person). Adapt consultation timing around clinician schedules.
Month 4 – "Consult / Collaborate" (Phase 2)	Build consensus and establish shared ownership	• All stakeholder groups<br>• PPI partner<br>• Advisory group	• Host co-design consensus workshop (consider using nominal group technique) to prioritise training topics.<br>• Identify "training champions" (early adopters).<br>• Audit engagement diversity and	In-person, hybrid or virtual	4 weeks (Weeks 13–16)	• Venue + catering<br>• Facilitation team<br>• Participant vouchers<br>• Data	Rotate facilitation between stakeholder groups to ensure equity.

			satisfaction.<br>• Share feedback summaries.			analysis support	Capture local examples to inform national recommendatio ns.
Month 5 – “Support”	Engage early implemente rs and begin capacity building	• Training champions<br>• Rehabilitation educators<br>• Technical staff<br>• PPI partner	• Identify small pilot funding opportunities and technical / pedagogical support needs.	Document	4 weeks (Weeks 17–20)	• Coordinator	Consider identified training priorities and how they fit with funding calls or pilot training opportunities..
Month 6 – “Dissemina te / Advocate”	Share results, prompt reflection, and encourage action	• All stakeholders<br>• NIHR HealthTech Centre<br>• Health educators<br>• Partner organisations<br>• Champions<br>• PPI partner	• Produce and circulate engagement summaries and/or infographics.<br>• Submit abstract(s) to conference.<br>• Draft manuscript(s) with PPI co-author.<br>• Hold dissemination webinar	Virtual dissemination, publications, HRC newsletter and events	4 weeks (Weeks 21–24)	• Comms support<br>• Graphic design (optional)<br>• Webinar hosting<br>• PPI time	Tailor dissemination format (infographics, summaries) for different audiences (patients, clinicians, innovators). Include local case examples.

Local adaptation:

- For rural or remote areas, rely more on virtual workshops and asynchronous methods.
- For low digital literacy stakeholders, offer hybrid formats or in-person sessions.
- Translate materials or provide accessibility accommodations (e.g. large print, captioning).
- Adjust timings around clinical workloads (e.g. evenings, weekends) or caregiver availability.

Also designate owners (which team members lead which strategies), and allocate budget (where available from Rehab HealthTech), resources (venues, travel, software) and contingency plans.

5. Evaluation and Outcomes (I-STEM Step 6)

Metrics and methods are required to evaluate whether the stakeholder engagement is succeeding (summative and formative). Outcomes and measurement plans are specified below.

Outcome	Definition / indicator	Measurement method	Timing/frequency	Purpose/rationale
**Acceptability**	Stakeholder rating of engagement process	Post-activity surveys (closed and open-ended items)	Immediately after each activity	To gauge how well stakeholders felt engaged and respected
**Adoption / enrolment**	Number of stakeholders who commit to attending activities	Attendance records	At each activity	To track stakeholder uptake and willingness to participate
**Appropriateness / perceived relevance**	Stakeholder assessment of fit between event summary (consensus priorities) and their own training priorities	Survey and/or discussion using checklist.	After each summary of stakeholder event	To check that stakeholder input is perceived to be relevant and useful to the final priority-setting.
**Feasibility/implementation fidelity**	How closely engagement strategies were delivered as planned	Logs and process tracking	Ongoing with mid-point check.	To monitor whether engagement is being delivered as intended.
**Stakeholder knowledge change**	Degree to which stakeholders' understanding of training possibilities has increased.	Survey and/or discussion using checklist.	After co-design activities.	To detect any shifts in stakeholder perspectives.

**Sustainment / embeddedness**	Whether stakeholder groups indicate interest in engaging with future training options	Survey and/or discussion using checklist.		

Summary

This I-STEM–aligned stakeholder engagement strategy provides a structured framework including objectives, stakeholder mapping, engagement approaches, strategies, concrete plan, and evaluation metrics. This will ensure that education and training interventions for rehabilitation technologies are co-created, relevant, and implementable. The inclusive, iterative design will enhance trust, ensure the voices of clinicians, patients, and innovators are heard, and foster long-term capacity for technology-enabled rehabilitation practice.
